# Supplementary material for: Using paired serology and surveillance data to quantify dengue transmission and control during a large outbreak in Fiji
Source: eLife. 2018 Aug 14;7:e34848. doi: 10.7554/eLife.34848 (PMC6092126; doi:10.7554/eLife.34848)
Supplement: Supplementary file 1. [file elife-34848-supp1.pdf]

Supplementary File 1

Health-seeking behaviour	Negative	% (95% CI)	Positive	% (95% CI)
No fever in preceding two years	77	79.4 (70-86.9)	26	68.4 (51.3-82.5)
Fever in preceding two years without visiting doctor	4	4.12 (1.13-10.2)	2	5.26 (0.644-17.7)
Visited doctor with fever in preceding two years	16	16.5 (9.73-25.4)	10	26.3 (13.4-43.1)
Total	97		38	

Supplementary File 1A: Relationship between seroconversion and health-seeking behaviour for participants who were initially seronegative by ELISA in 2013. Table shows breakdown for participants who were also negative in 2015 (i.e. did not seroconvert) and positive in 2015 (i.e. seroconverted).

Assay	Year	DENV-1	DENV-2	DENV-3	DENV-4	Any DENV
ELISA	2013	-	-	-	-	64% (49.2-77.1%)
	2015	-	-	-	-	92% (80.8-97.8%)
MIA	2013	64% (49.2-77.1%)	16% (7.17-29.1%)	38% (24.7-52.8%)	34% (21.2-48.8%)	72% (57.5-83.8%)
	2015	86% (73.3-94.2%)	18% (8.58-31.4%)	72% (57.5-83.8%)	50% (35.5-64.5%)	92% (80.8-97.8%)
NT	2013	64% (49.2-77.1%)	44% (30-58.7%)	44% (30-58.7%)	30% (17.9-44.6%)	74% (59.7-85.4%)
	2015	84% (70.9-92.8%)	64% (49.2-77.1%)	90% (78.2-96.7%)	52% (37.4-66.3%)	96% (86.3-99.5%)

Supplementary File 1B: Proportion seropositive against each of the four DENV serotypes and any DENV (i.e. at least one serotype) for a subset of 50 individuals tested using ELISA, MIA and neutralisation assays.

Parameter	SEIR	SEIR + climate	SEIR + climate + control
$1/\nu_h$	4.6 (2.9-6.7)	5.3 (4.1-6.8)	5.9 (4.6-7.5)
$1/\gamma$	3.9 (2.8-5.3)	4.8 (3.6-6.1)	5.3 (3.9-6.9)
$1/\hat{\nu_v}$	9.7 (8-12)	9.8 (7.9-12)	9.8 (8-12)
$1/\hat{\delta}$	6.3 (3.7-9)	7.5 (6-9.2)	8.7 (6.8-11)
$\hat{\alpha}$	0.2 (0.12-0.4)	0.16 (0.057-0.59)	0.43 (0.19-1)
$\hat{m}$	16 (11-20)	5.4 (0.4-43)	0.69 (0.14-3.5)
$\hat{K}$	2.4 (1.1-7.9)	10 (0.69-83)	34 (6.5-96)
a_1	8.8 (1.7-120)	0.47 (0.054-350)	55 (6.2-450)
a_2	0.18 (0.057-0.91)	0.15 (0.028-0.86)	0.58 (0.32-0.83)
a_τ	28 (0.038-28)	0.0021 (0.0013-27)	0.0017 (0.0013-0.0096)
r_{lab}	0.074 (0.038-0.16)	0.11 (0.074-0.19)	0.13 (0.086-0.2)
r_{DLI}	0.11 (0.045-0.25)	0.12 (0.073-0.25)	0.11 (0.073-0.19)
ρ	1.4 (0.43-2.1)	0.87 (0.62-1.3)	0.57 (0.38-0.88)
I_{hc}^0	10 (3.9-20)	1700 (130-4400)	140 (18-550)
I_{ha}^0	2.9 (0.98-7.3)	0.32 (0.0077-64)	130 (19-680)
R_{hc}^0	0.056 (0.029-0.097)	0.083 (0.04-0.15)	0.064 (0.027-0.12)
R_{ha}^0	0.4 (0.32-0.47)	0.42 (0.36-0.49)	0.43 (0.36-0.49)
I_v^0	0.0026 (0.00024-0.0069)	0.00031 (1.6e-05-0.0059)	1e-04 (1.2e-05-0.0015)

Supplementary File 1C: Posterior distributions for parameters in Table 7 using models fitted to surveillance data and MIA data.

Parameter	SEIR	SEIR + climate	SEIR + climate + control
$1/\nu_h$	5 (3.5-6.9)	5.1 (3.9-6.7)	5.7 (4.4-7.2)
$1/\gamma$	4 (2.7-5.8)	4.6 (3.4-5.8)	5 (3.7-6.3)
$1/\hat{\nu_v}$	9.6 (7.7-12)	9.6 (7.9-12)	9.7 (7.9-12)
$1/\hat{\delta}$	6.6 (4.5-9.1)	7 (5.7-8.9)	8 (6.4-10)
$\hat{\alpha}$	0.25 (0.12-0.51)	0.24 (0.14-0.33)	0.42 (0.15-0.63)
$\hat{m}$	26 (1.8-49)	7.2 (4.3-21)	1.7 (0.74-12)
$\hat{K}$	0.096 (0.02-0.92)	2.4 (0.51-5.5)	11 (2.3-66)
a_1	1.4 (0.021-890)	420 (110-980)	57 (12-890)
a_2	0.023 (0.0011-0.68)	0.26 (0.12-0.66)	0.68 (0.38-0.93)
a_τ	28 (28-28)	0.0094 (0.0022-25)	0.0013 (0.0013-0.27)
r_{lab}	0.11 (0.056-0.8)	0.081 (0.062-0.11)	0.14 (0.094-0.23)
r_{DLI}	0.18 (0.066-1.4)	0.083 (0.059-0.11)	0.13 (0.087-0.2)
ρ	1.2 (0.4-2.1)	0.37 (0.24-0.58)	0.31 (0.19-0.49)
I_{hc}^0	16 (3.2-2700)	41 (16-250)	0.98 (0.21-3.8)
I_{ha}^0	0.43 (0.015-2.8)	42 (7.8-110)	1.3 (0.0094-57)
R_{hc}^0	0.21 (0.13-0.31)	0.19 (0.13-0.27)	0.21 (0.14-0.3)
R_{ha}^0	0.74 (0.66-0.81)	0.73 (0.66-0.78)	0.75 (0.69-0.8)
I_v^0	0.0019 (0.00012-0.02)	0.00048 (0.00026-0.0012)	0.00039 (0.00014-0.001)

Supplementary File 1D: Posterior distributions for parameters in Table 7 using models fitted to surveillance data and ELISA data.
