# Supplementary material for: Using paired serology and surveillance data to quantify dengue transmission and control during a large outbreak in Fiji
Source: eLife. 2018 Aug 14;7:e34848. doi: 10.7554/eLife.34848 (PMC6092126; doi:10.7554/eLife.34848)
Supplement: Supplementary file 2. [file elife-34848-supp2.pdf]

Data collection form

Part A: Participant information

(Selecting individuals both agreeing further sample testing and further contact)

Participant ID FJD CP☐☐☐☐

First name:________________________ **Family Name :** ___________________________________ **Age:** ___________

Part B: Visit record sheet

Date today: __ __ / __ __ / 20__ __ (dd/mm/yyyy)

Interviewer: ____

Bula vinaka! Thank you for agreeing to take part in this research. We would like to ask you a few questions to help us understand dengue fever in Fiji. Please stop me and ask questions whenever you would like.

Have **you** had a fever in the last 2 years?
Circle: 1=yes 0=no, -1=don't know, -2=refused

Have **you** visited a doctor with a fever in the last 2 years?
Circle: 1=yes 0=no, -1=don't know, -2=refused

If YES to both	Fever episode (or 1st episode if more than one)	2nd episode (if more than one)	3rd episode (if more than two)
When? Year (-1=don't know)			
Month, if known (-1=don't know)			
Did the doctor suspect it was dengue fever? 1=yes, 0=no, -1=don't know, -2=refused			
Did the doctor do a blood test for dengue fever? 1=yes, 0=no, -1=don't know, -2=refused			
Was the blood test positive for dengue fever? 1=yes 0=no, -1=don't know, -2=refused			
Were you hospitalised? 1=yes 0=no			

Have **any household members** visited a doctor with a fever in the last 2 years?			
Circle: 1=yes 0=no, -1=don't know, -2=refused			
If YES, give details of most recent episode (for up to 3 household members)			
	Member 1	Member 2	Member 3

That person's age now			
When? Year (-1=don't know)			
Month, if known (-1=don't know)			
Did the doctor suspect it was dengue fever? 1=yes, 0=no, -1=don't know, -2=refused			
Did the doctor do a blood test for dengue fever? 1=yes, 0=no, -1=don't know, -2=refused			
Was the blood test positive for dengue fever? 1=yes 0=no, -1=don't know, -2=refused			
Were they hospitalised? 1=yes 0=no 1=don't know, -2=refused			

Presence or absence at the house of:

**Item**	Yes	No	Could not find out
Mosquitoes			
Used car tires			
Open water containers (e.g. vase, bucket, oil drum)			
Air conditioning			
Blocked ditches/drains			
Other mosquito breeding grounds<br>If yes, specify________________________			

Check new telephone number if not previously recorded. Add new address GPS, if moved house. Check: is date of move recorded?

GPS coordinates of 1= front door of house, 2= community centroid,
South 0□□°□□.□□□’ (circle) **East** □□□°□□.□□□’

Any other comments?

Vinaka vaka levu.
